# Prognostic value of pretreatment systemic inflammatory markers in patients with stage I endometrial cancer

**DOI:** 10.7150/ijms.78182

**Published:** 2022-11-07

**Authors:** Jung Hwan Ahn, Sung Jong Lee, Joo Hee Yoon, Dong Choon Park, Sang Il Kim

**Affiliations:** 1 Department of Obstetrics and Gynecology, Yonsei University Wonju College of Medicine, Republic of Korea.; 2 Department of Obstetrics and Gynecology, Seoul St. Mary's Hospital, College of Medicine, The Catholic University of Korea, Seoul, Republic of Korea.; 3 Department of Obstetrics and Gynecology, St. Vincent's Hospital, College of Medicine, The Catholic University of Korea, Seoul, Republic of Korea.

**Keywords:** endometrial cancer, uterine cancer, systemic inflammation reponse, adverse risk factors, monocyte-lymphocyte ratio

## Abstract

**Objective:** Evaluate the prognostic value of monocyte-lymphocyte ratio (MLR) in patients with stage I endometrial cancer.

**Method:** Data from 225 patients with stage I endometrioid endometrial cancer who underwent surgical resection between January 2010 and December 2020 were reviewed. The receiver operating characteristic (ROC) curves were generated for the neutrophil-lymphocyte ratio, platelet-lymphocyte ratio, and MLR. Optimal cut-off values were determined as the points at which the Youden index (sensitivity + specificity - 1) was maximal. Based on the results of the ROC curve analysis, the patients were grouped into high MLR and low MLR groups. Recurrence rate, and disease-free survival were compared between the two groups. The prognostic factors were investigated using univariate and multivariate Cox proportional hazards model.

**Results:** The optimal cut-off value of MLR was 0.220 (AUC, 0.835; *p* < 0.001). Significantly more patients in the high MLR group experienced recurrence (20.3% vs. 1.9%, *p* < 0.0001). In multivariate analysis, grade, depth of myometrial invasion, adjuvant RT, and high MLR were independent prognostic factors for disease-free survival.

**Conclusion:** Elevated MLR was significantly associated poor clinical outcomes in patients with stage I endometrioid endometrial cancer. Our findings suggest that MLR may be clinically reliable and useful as an independent prognostic marker for patients with stage I endometrioid endometrial cancer.

## Introduction

Endometrial cancer (EC) is the most common gynecologic cancer affecting women in developed countries 1. Approximately 65,950 new cases and 12,550 deaths related to EC are expected to occur in the United States in 2022 2.

About 70% of patients with endometrioid EC were diagnosed with stage I disease, and 5-year survival rate was nearly 90% 2. The primary standard treatment for stage I endometrioid EC is surgery. After surgery, adjuvant treatment is recommended based on the patient's adverse risk factors 3. Traditional prognostic factors for EC include initial stage, grade, histologic subtype, age at diagnosis, tumor size, and lymphovascular space invasion (LVSI) 4-6. However, since stage I endometrioid EC has an excellent prognosis with a low rate of recurrence, these conventional risk factors are not sufficiently accurate to predict survival outcomes. A small but substantial number of patients with stage I endometrioid EC experience recurrence of disease and poor survival 7. Thus, novel approaches for pre-treatment assessment to identify probable recurrence are crucial.

Peripheral blood cells, including neutrophils, lymphocytes, and monocytes, are biomarkers of tumor immunity and can reflect the cancer-related inflammatory microenvironment 8. Earlier studies have reported that systemic inflammatory responses play important roles in carcinogenesis, progression, and prognosis 9-11. The neutrophil-lymphocyte ratio (NLR), platelet-lymphocyte ratio (PLR), and monocyte-lymphocyte ratio (MLR) are the currently available markers of the systemic inflammatory response 12. These markers have been clarified to show prognostic significance in solid cancers, including gynecologic cancer 13-18. However, the prognostic value of these ratios in patients with stage I endometrioid EC is unclear. Therefore, this study aimed to evaluate the prognostic value of NLR, PLR, and MLR for patients with stage I endometrioid EC.

## Materials and Methods

This retrospective study was approved by the Institutional Review Board of the Catholic University of Korea. The requirement for informed consent was waived owing to the nature of the study. The study was conducted in accordance with the principles of the Declaration of Helsinki.

We reviewed our institution's cancer registry and identified patients who underwent primary surgical treatment for EC from January 2010 to December 2020. The retrospective review included all patients who were diagnosed as having EC. Thus, data from 338 patients were recorded in a single database.

We excluded patients who did not receive primary surgery; showed non-endometrioid histology; had stage II, III, or IV disease, inflammatory disease, hematological disease, or autoimmune disease; or had no preoperative complete blood cell count data or complete blood cell count data obtained within 2 weeks before surgery. Patients with incomplete clinicopathological or follow-up data were also excluded. The remaining 225 patients were included as the study population.

All patients underwent total hysterectomy, bilateral salpingo-oophorectomy, and systematic lymphadenectomy. Systemic lymphadenectomy included pelvic and para-aortic lymphadenectomy; however, the latter could be omitted when the pelvic lymph nodes were disease-free. Postoperatively, patients were treated with adjuvant radiation therapy according to the disease risk factors.

NLR and PLR were defined as the absolute neutrophil count and platelet count, respectively, divided by the absolute lymphocyte count. Similarly, MLR was defined as the absolute monocyte count divided by the absolute lymphocyte count. Disease-free survival (DFS) was measured from the date of diagnosis of EC to the date of first disease progression. If the patient showed no recurrence, the observation was censored at the date of death or the last follow-up. Overall survival (OS) was measured from the date of initial diagnosis to the date of cancer-related death or the last follow-up. The primary endpoint was DFS, and the secondary endpoint was OS.

Receiver operating characteristic (ROC) curves of DFS were generated for NLR, PLR, and MLR. The optimal cut-off values of NLR, PLR and MLR were determined as the points at which the Youden index (sensitivity +specificity - 1) values were maximal. Based on the results of ROC curve, patients were divided into high-MLR and low-MLR group. DFS and OS were analyzed by the Kaplan-Meier method, and the curves were compared using the log rank test. We performed univariate and multivariate analyses using Cox proportional hazards model to evaluate effects of prognostic factors. All statistical analyses were performed using Statistical Package for the Social Science (SPSS) statistical software package, version 22.0 (SPSS Inc., Chicago, IL, USA), and *p* < 0.05 was considered statistically significant.

## Results

Overall, 225 patients were included in the final analysis. The baseline characteristics of the patients are presented in Table 1.

**Figure 1:**
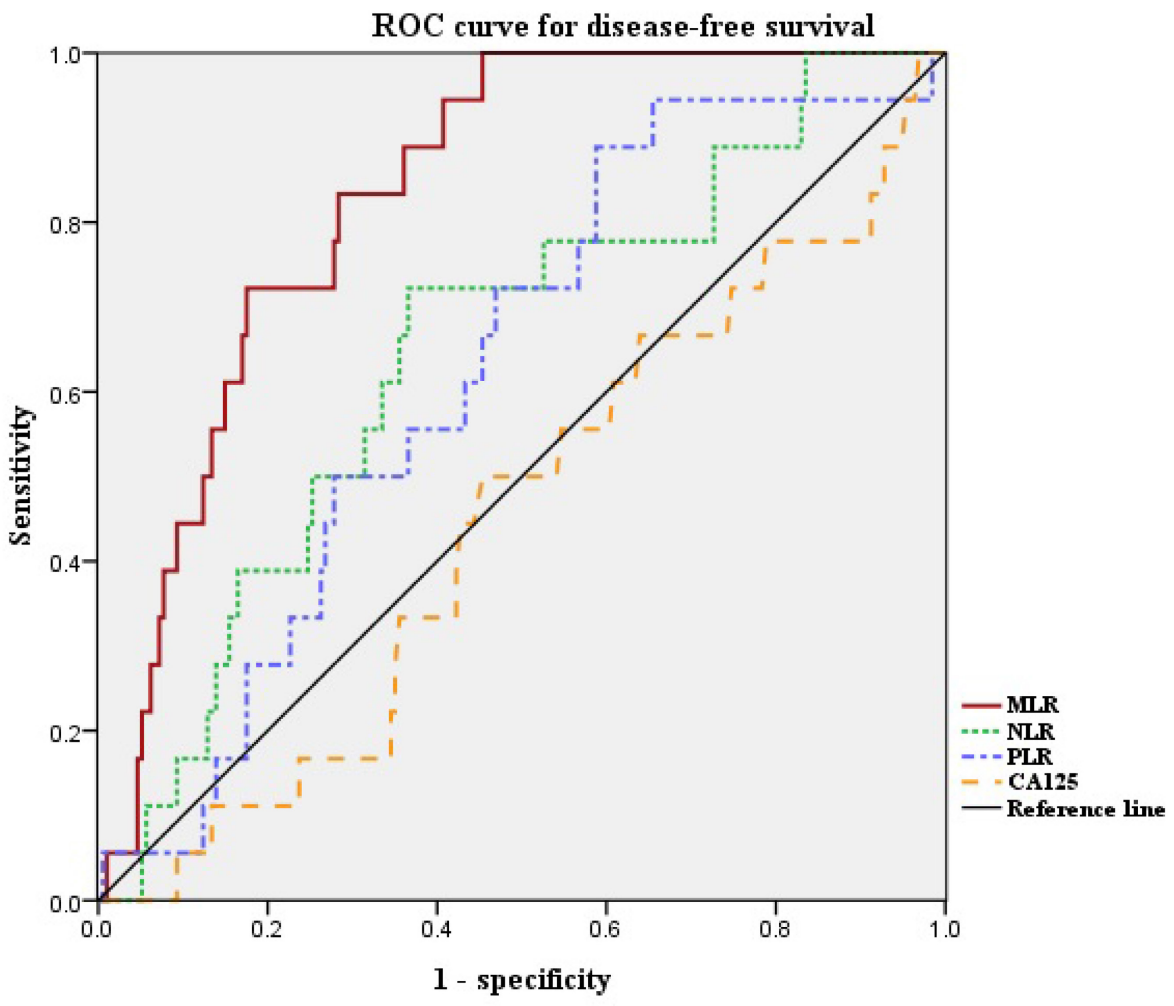
ROC curves for DFS of NLR, PLR and MLR. Optimal NLR, PLR and MLR cut-off value was 1.889, 117.3 and 0.220 respectively. The AUC was 0.653, 0.637 and 0.835. ROC, receiver operating characteristic; AUC, area under the curve; NLR, neutrophil-to-lymphocyte ratio; PLR, platelet-to-lymphocyte ratio; MLR, monocyte-to-lymphocyte ratio.

Next, we defined the optimal cut-off values of NLR, PLR, and MLR by ROC curve analysis for our patient population (Figure 1). The median NLR was 1.626 (range, 0.611-6.417). The optimal cut-off value of NLR for DFS was 1.889 (area under the curve [AUC]: 0.653; 95% confidence interval [CI]: 0.526-0.780, *p* = 0.07). Median PLR was 131.7 (range, 37.4-349.6). The optimal cut-off value of PLR for DFS was 117.3 (AUC: 0.637; 95% CI: 0.521-0.753, *p* = 0.09). Median MLR was 0.191 (range, 0.037-0.755). The optimal cut-off value of MLR for DFS was 0.220 (AUC: 0.835; 95% CI: 0.764-0.906, *p* < 0.001). The ROC curve analysis suggested that the AUC of MLR was the highest, and the MLR was the only marker to show statistical significance. Thus, we divided the patients into high-MLR (MLR ≥ 0.220) and low-MLR (MLR < 0.220) group.

The associations between MLR and clinicopathologic factors are presented in Table 2. The low- and high-MLR groups included 151 (67.1%) and 74 (32.9%) patients, respectively, with no statistically significant differences between the two groups in terms of age, body mass index (BMI), grade, depth of myometrial invasion (MMI), tumor size, LVSI status, and adjuvant treatment after surgery. Interestingly, significantly more patients in the high-MLR group experienced recurrence (20.3% vs. 1.9%, *p* < 0.001).

To evaluate the prognostic factors of recurrence, we used Cox's proportional hazards model (Table 3).

Univariate analysis revealed that DFS was significantly associated with grade, MMI, adjuvant radiotherapy (RT) and MLR. LVSI status and tumor size were not associated with DFS. Moreover, other inflammatory markers, such as NLR and PLR, were not associated with DFS either. In multivariate analysis, grade, MMI, adjuvant RT, and high MLR were independent prognostic factors for DFS.

**Figure 2:**
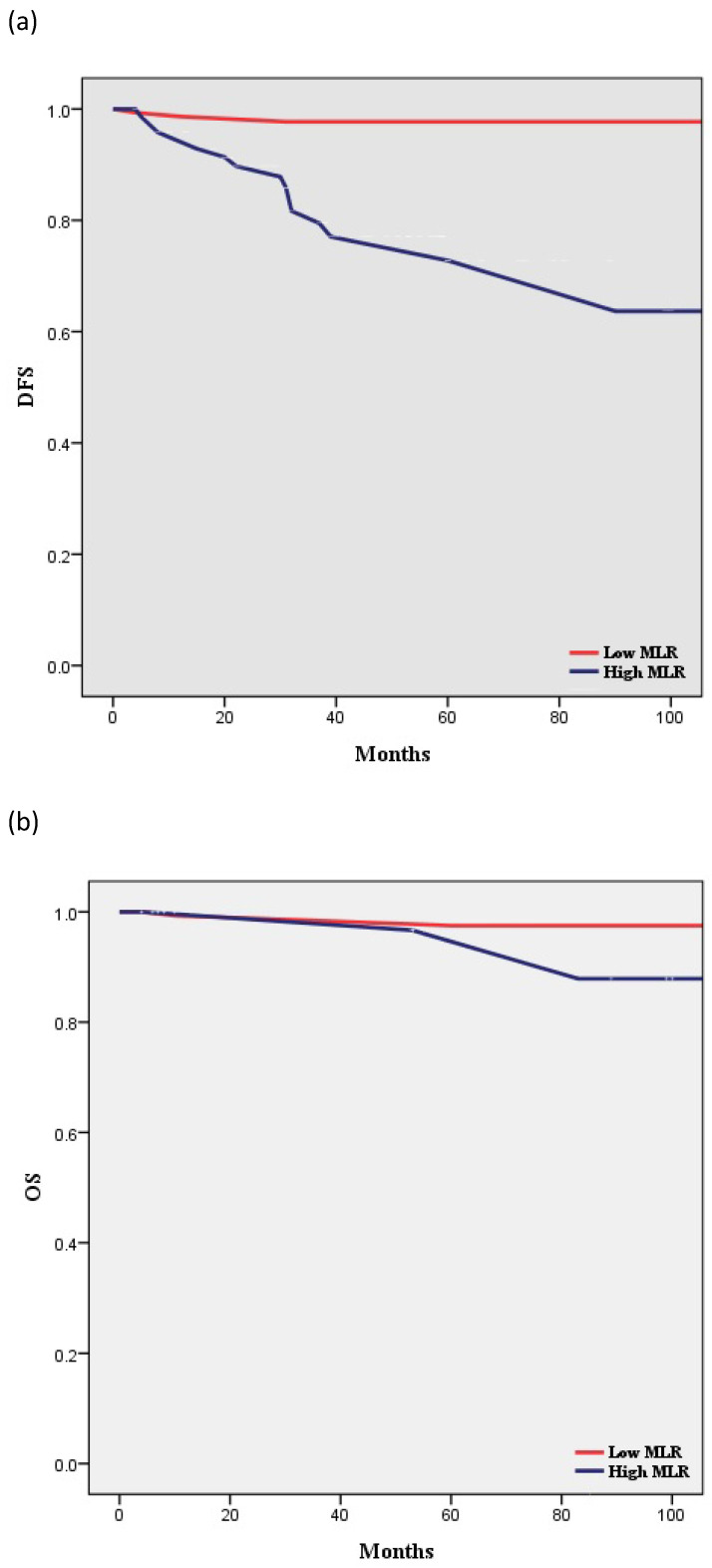
Survival curves according to MLR: (**a**) Kaplan-Meier survival curves for DFS of patients with a high MLR and those with a low MLR (HR, 15.56; 95% CI, 5.521-43.83; P < 0.001). (**b**) Kaplan-Meier survival curves for OS of patients with a high MLR and those with a low MLR (HR, 2.509; 95% CI, 0.298-21.11; P = 0.397). DFS, disease-free survival; OS, overall survival; MLR, monocyte-to-lymphocyte ratio.

According to Kaplan-Meier analysis, the 5-year DFS rates in the low- and high-MLR groups were 97.7% and 63.7% (*p* < 0.001), respectively, and the 5-year OS rates in these two groups were 97.5% and 96.7%, respectively (*p* = 0.397) (Figure 2). The two groups showed no statistically significant differences in terms of OS.

## Discussion

The association between inflammation and cancer was first described by Virchow in 1863 19. Since then, numerous studies have highlighted the importance of inflammatory cells and cytokines, which are more likely to contribute to tumor growth, progression, and metastasis 20, 21. These findings indicate that a systemic inflammatory response is a basic feature of malignancy. Moreover, previous studies reported the association between the systemic inflammatory response and the prognosis of solid tumors, including gynecologic cancer 13-18.

The majority of endometrioid EC patients are diagnosed as showing stage I disease. Most of the stage I endometrioid EC patients were treated with surgery alone, but adjuvant treatment is recommended in patients with adverse risk factors. Pathologic factors that may influence the decision regarding adjuvant therapy include LVSI, grade, tumor size, and depth of invasion 4-6. Patients without adverse risk factors are defined as showing low-risk EC. The low-risk group represents the largest group of patients with stage I EC and presents excellent survival outcomes 28. However, 3%-10% of these patients experience relapse 3. Thus, identification of novel indicators is essential to ensure prompt detection of probable recurrence. In the present study, the MLR was demonstrated as a surrogate marker for DFS in multivariate analysis. These results are in concordance with previous studies in which the MLR was suggested to be associated with survival in patients with endometrial cancer, ovarian cancer, colorectal cancer, and hepatocellular carcinoma 23-26. Thus, the findings of this study indicate that the preoperative MLR was an independent predictor of recurrence in patients with stage I endometrioid EC, including low-risk EC, and the results provide a valuable clue for evaluation of the systemic inflammatory response to predict the recurrence of low-risk EC.

The precise mechanisms of the association between a high MLR and poor outcomes have not been clarified. The MLR is thought to reflect the balance between the unfavorable role of monocytes and the favorable prognostic effect of lymphocytes 27. Monocytes are known to have pro-tumoral functions, such as differentiation into tumor-associated macrophages (TAMs), metastatic cell seeding, suppression of T cell function, angiogenesis, and extracellular matrix remodeling 28. TAMs accelerate tumor progression and invasion by releasing growth factors and angiogenic factors 29. Lymphocytes, in contrast, are usually known for their anti-tumor functions, which include induction of apoptosis and suppression of proliferation 8. CD8^+^ T lymphocytes attack tumor cells via cytotoxicity, while CD4^+^ T lymphocytes exhibit potent anti-tumor immune response 30. Thus, a low lymphocyte count and high monocyte count might be associated with cancer progression and a poor prognosis. An elevated MLR can be attributed to a relative increase in the monocyte count or relative decrease in lymphocyte count. Thus, the MLR may serve as a surrogate marker reflecting increased cancer aggressiveness.

The NLR and PLR have also been suggested to be related to cancer patient prognosis. A higher NLR and PLR have been shown to be associated with poor prognosis in patients with EC 31, 32. However, in our study, higher NLR and higher PLR were not associated with poor survival.

Recent study by *Crosbie* et al. suggested C-reactive protein (CRP) as a prognostic biomarker in EC patients 33. In this study, MLR was associated with adverse factors, but not overall, cancer-specific or recurrence-free survival in the multivariable analysis. A different conclusion might be reached based on alternative thresholds, since there are no clinically validated prognostic thresholds for MLR.

The adverse risk factors in patients with stage I EC include high grade, deep MMI, LVSI, and tumor size 34, 35. Stratification of patients for adjuvant RT is based on these factors. In our study, adverse risk factors such as LVSI and large tumor size were not associated with survival outcomes while patients who received adjuvant RT had favorable outcomes. Thus, our findings highlight the potential benefit of RT in patients at increased risk of recurrence, especially LVSI and large tumor size.

The histologic grade of EC is an important factor associated with its prognosis. The majority of low-grade ECs tend to limit their spread to the surface of the endometrium, with a low likelihood of disease extension beyond the uterine corpus or the need for adjuvant therapy 36. In our study, a higher grade was associated with an increased risk of recurrence. Interestingly, not only grade 3, but grade 2 EC was also associated with an increased risk of recurrence. All patients with grade 3 EC received adjuvant RT. However, patients with grade 2 EC received adjuvant RT only if they had additional risk factors such as deep MMI, LVSI, or a large tumor size. Thus, our findings indicate that not only grade 3, but grade 2 EC is also adverse risk factors in patients with stage I EC.

Our study had several limitations. First, it was a retrospective single-center study. Second, the number of enrolled patients was small. These results need to be confirmed in a large cohort. Third, since there was no defined MLR value, we had to set a cut-off value for our population.

In conclusion, we found that an elevated MLR was significantly associated with a lower DFS in stage I endometrioid EC patients. Our findings suggest that the MLR may be clinically reliable and useful as an independent prognostic marker for stage I endometrioid EC patients. Further prospective studies are needed to confirm our findings and to identify appropriate cut-off values.

## Data Availability

The data that support the findings of this study are available on request from the corresponding author.

## Figures and Tables

**Table 1:** Baseline patient characteristics (n = 225).

Patient characteristics	Stage I EC (n=225)
Age (years^ *^	54 (28 - 81)
BMI (kg/m^2 *^	24.9 (15.2 - 39.3)
Grade^+^	
1	126 (56.0)
2	72 (32.0)
3	27 (12.0)
Myometrial invasion^+^	
< 50%	188 (83.6)
≥ 50%	37 (16.4)
Tumor size (cm)^*^	2.4 (0 - 10.0)
LVSI	
Absent	207 (92.0)
Positive	18 (8.0)
Adjuvant radiotherapy^+^	
No	162 (72.0)
Yes	63 (28.0)
Median follow-up (months)^*^	46 (4 - 144)
Overall recurrences^+^	18 (8.0)
Deaths^+^	4 (1.8)

^*^median (range), ^+^number (percent). EC, endometrial cancer; BMI, body mass index; LVSI, lymphovascular space invasion.

**Table 2:** Clinical and pathological characteristics according to the MLR (n=225).

	Low MLR group (n = 151)	High MLR group (n = 74)	*p* value
Age (years)^*^	56 (32 - 78)	53 (28 - 81)	0.598
BMI (kg/m^2^)^*^	24.8 (18.5 - 37.8)	25.1 (15.2 - 39.3)	0.201
Grade^+^			0.131
1	78 (51.6)	48 (64.9)	
2	54 (35.8)	18 (24.3)	
3	19 (12.6)	8 (10.8)	
Myometrial invasion^+^			0.848
< 50%	127 (84.1)	61 (82.4)	
≥ 50%	24 (15.9)	13 (17.6)	
Tumor size (cm)^*^	2.4 (0 - 10.0)	2.5 (0 - 7.0)	0.968
LVSI^+^			0.577
Absent	139 (92.1)	68 (91.9)	
Positive	12 (7.9)	6 (8.1)	
Adjuvant RT^+^			0.136
No	104 (68.9)	58 (78.4)	
Yes	47 (31.1)	16 (21.6)	
Recurrence^+^	3 (1.9)	15 (20.3)	< 0.001
Death^+^	2 (1.3)	2 (2.7)	0.397

^*^median (range), ^+^number (percent). MLR, monocyte-to-lymphocyte ratio; BMI, body mass index; LVSI, lymphovascular space invasion; RT, radiotherapy.

**Table 3:** Univariate and multivariate analysis of prognostic factors for disease-free survival (n = 225).

Characteristics	Univariate analysis			Multivariate analysis		
	OR	95% CI	*p value*	OR	95% CI	*p value*
Grade						
1	1 (Ref)	-	-	1 (Ref)	-	-
2	24.517	5.005 - 120.097	0.001^*^	25.272	5.787 - 110.354	0.001^*^
3	26.053	3.678 - 184.560	0.002^*^	24.026	4.331 - 133.270	0.001^*^
MMI						
< 50%	1 (Ref)	-	-	1 (Ref)	-	-
≥ 50%	7.440	1.224 - 45.230	0.029^*^	9.093	2.166 - 38.177	0.003^*^
LVSI						
Absent	1 (Ref)	-	-			
Positive	0.686	0.038 - 12.436	0.799			
Tumor size						
< 2cm	1 (Ref)	-	-			
≥ 2cm	1.542	0.304 - 7.834	< 0.601			
Adjuvant RT						
No	1 (Ref)	-	-	1 (Ref)	-	-
Yes	0.068	0.008 - 0.551	0.012^*^	0.063	0.012 - 0.339	0.001^*^
NLR						
< 1.889	1 (Ref)	-	-			
≥ 1.889	2.569	0.594 - 11.116	0.207			
PLR						
< 117.3	1 (Ref)	-	-			
≥ 117.3	2.050	0.327 - 12.848	0.443			
MLR						
< 0.220	1 (Ref)	-	-	1 (Ref)	-	-
≥ 0.220	23.928	5.594 - 102.349	< 0.001^*^	20.643	5.616 - 75.873	0.001^*^

Covariates with *p* < 0.05 on univariate analysis were included in multivariate model. OR, odds ratio; CI, confidence interval; Ref, reference; MMI, myometrial invasion; LVSI, lymphovascular space invasion; RT, radiotherapy; NLR, neutrophil-to-lymphocyte ratio; PLR, platelet-to-lymphocyte ratio; MLR, monocyte-to-lymphocyte ratio.
